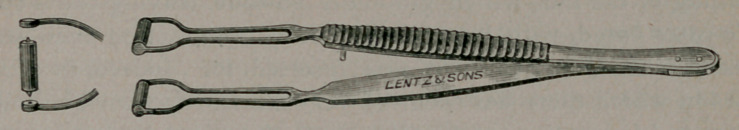# The Mechanical Treatment of Trachoma

**Published:** 1892-08

**Authors:** Edward Jackson

**Affiliations:** Professor of Diseases of the Eye in the Philadelphia Polyclinic, Surgeon to Wills Eye Hospital, etc.


### DANIEL'S TEXAS MEDICAL JOURNAL.

ESTABLISHED JULY, 1885.

PUBLISHED MONTHLY.—SUBSCRIPTION $2.00 A YEAR.

Vol. VIII. AUSTIN, AUGUST, 1892. No. 2.

### Original Contributions.

For Daniel's Texas Medical Journal.

### THE MECHANICAL TREATMENT OF TRACHOMA.

BY EDWARD JACKSON, M. D.,
Professor of Diseases of the Eye in the Philadelphia Polyclinic, Surgeon to Wills Eye Hospital, etc.

THE importance and dangers of trachoma, or true granular conjunctivitis, as a cause of suffering and blindness, and its spread by contagion, were well presented by Dr. J. A. Lippincott, in the address on ophthalmolgy, last year. An important fact in its transmission, is its extremely chronic course. I have in mind a case which I saw and treated, from time to time, for ten years, the patient attending for a time, then getting weary and neglecting treatment for awhile, and anon returning, each time with the cornea distinctly more damaged; and during the whole of this period this patient was a possible center of infection to those around her. And such cases are not exceptional, but extremely common.

It is rather exceptional to have a patient persevere steadily with treatment until the cure is complete. Last year I remember but a single case, in my service at Wills Hospital, who persisted steadily to the end, coming much of the time three times a week for applications of copper sulphate, and who was finally discharged completely cured, just about a year after the treatment

NOTE—Advance proof from Transactions.

was commenced. There is still attending my service at the Polyclinic Hospital, for occasional applications, a girl who has been under pretty steady treatment with astringents for four years, and who is now about cured, with some shrinkage of the conjunctiva. And during these protracted periods we have to deal not with sequellæ, but with the active disease running its characteristic course. I cannot recall seeing a case of well-marked trachoma, even when taken early, that was thoroughly cured by applications of copper, alum, silver, and tannin, in less than three months. Yet such treatment has been, and still is, mainly relied on, for the radical cure of this disease.

In contra distinction to this, attention should be called to the results of mechanical treatment. Many attempts have been made in this direction, such as incising the trachoma granules, the little sago-like and gray translucent masses that crowd the retro-tarsal fold and are scattered over the palpebral portion of the conjunctiva. Others have excised them, sometimes removing the whole retro-tarsal fold, greatly abridging the disease, but also leaving a contracted conjunctival sac.

Others, led by Manolescu, had resorted to grattage, or brossage, scraping the whole conjunctival surface, or brushing it with a tooth brush, the bristles of which were cut short to stiffen them, with or without the application of strong solutions of mercuric chloride. This latter method, if thoroughly applied, is effective in promptly cutting short the disease; but leaves such cicatricial contraction of the lids that its adoption seems scarcely justifiable.

In 1886, Dr. C. F. Hotz published his method of expression, which it is since claimed had also been practised many years before by Sir William Wilde and others. He pressed out the granulations from the upper lid and cul-de-sac with the thumb-nail, and from the lower lid by the aid of iris forceps, from which the corrugations had been ground off. This operation, he urged, could be done without a general anæsthetic; and he claimed that it greatly shortened the treatment of the most tedious cases, those with well-marked trachoma granules. I tried it, as did doubtless many others, under cocaine anæsthesia, and, probably on account of failure to thoroughly express the contents of the granules, was not very much impressed by the method. Others, notably Drs. A. E. Prince and H. D. Noyes, devised forms of forceps for the operation, and practiced it with great success.

Something over a year ago, Dr. H. Knapp resorted to the roller principle, such as is applied to the common mangle or clothes wringer, to the expression of the trachoma granules, and devised a pair of roller forceps, here shown, for the purpose.

His results were extremely satisfactory, the mass of cases being cured by a single operation often without necessity for subsequent treatment, and without cicatrices due to the operation. He reported them at the meeting of the American Ophthalmological Society last September, and they have been so far confirmed in the experience of others who have tried the method, that it should be adopted for all suitable cases.

The method of performing the operation of expression with this instrument, is as follows:

The patient being fully etherized, one of the lids is everted. In everting the upper lid, I fold over the tarsal portion in the usual method of everting the lid, then seize the folded margin and withdraw it from the globe, at the same time everting it and fully exposing the upper cul-de-sac of the conjunctiva. The forceps are then thrust well into the cul-de-sac, and as large a fold of the conjunctiva as possible is firmly grasped between the rolls. Firm pressure is then made, and the rolls steadily withdrawn. As this is done, the material composing the trachoma granules in the portion of the conjunctiva so grasped is seen to ooze out from the tissues on the rolls until the fold escapes from the forceps freed of this morbid material. A second portion of the conjunctiva is then grasped and similarly stripped, and this is repeated throughout the conjunctival tract.

Especial care is to be given to the thorough cleansing of the parts of the membrane in the vicinity of the commissures, this being the most difficult part of the operation. The squeezing of the portion of the membrane lining the tarsal portion of the lids near their free margin is best accomplished, by placing one roll on the conjunctival and one roll on the dermal surface of the lid, and drawing them up to the free margin of the lid.

In this way, every portion of the conjunctiva is to be gone over two or three times, until no more of the morbid exudate can be squeezed out. The conjunctiva is then to be cleansed of blood and exudate, and the after-treatment is to be directed to prevent undue reaction at first, and subsequently to prevent relapses.

The effect of this operation is at first to cause œdema and swelling of the lids, which gradually subside, leaving the favorable cases cured, with no more cicatricial contraction than was present when this treatment was resorted to. Indeed, in those cases in which there has occurred very marked cicatricial thickening and induration of the lid, the induration disappears entirely, and while there is later a tendency to its return, the ultimate result appears to be a very marked permanent benefit.

The amount of benefit, however, is in general proportioned to the amount of the characteristic exudate present in and beneath the conjunctiva. Where this is the leading feature of the case, the cure is immediate, and, so far as I have been able to observe, permanent. But it is remarkable how much of this translucent, jelly-like matter can be pressed out of some thick indurated lids, that give very little evidence exteriorly of its presence.

In the favorable cases, those in which the granulations are well marked and the general induration and alteration of the deeper structures of the lid comparatively slight, a single rolling is sufficient. In the less favorable cases, those in which there is more induration and thickening, Dr. Knapp advises the free incision or scarification of the lid prior to rolling. In these cases, including the worst that I have encountered since resorting to this method of treatment, there was marked improvement after the operation, and it was repeated with still further improvement.

I have done the operation under cocaine anæsthesia, where the granulations were localized in a limited part of the conjunctiva, and the patient greatly preferred not to take ether. But the cases in which it is advisable to do it are quite exceptional, for without general anæsthesia the squeezing will not be thoroughly completed in every part of the conjunctiva, and it is only the thorough expression that produces the cures, which seem truly marvelous when compared with the results of older methods of treatment.

A word about the selection of the instrument; the essential point is that it should roll easily. This is first to be tested by seizing with it the fold of skin between the fingers, and noting if the rolls properly turn under very light pressure, and do not slide over the surface without turning. To ascertain if their motion is uniform, grasp a piece of paper firmly between the rolls and pull it out while firm pressure is maintained. If the rolls are at all irregular in outline, or not properly centered, the movement will be found decidedly jerky, instead of smooth and uniform, as it should be. In using the instrument, one must watch that it does not become clogged with blood and exudation and cease to roll, simply dragging over the tissues, for this does not effectually empty them of the trachomatous matter, and is liable to cause tearing of the conjunctiva.

The corrugated rolls were adopted by Dr. Knapp, I believe, for the purpose of more certainly securing their rotation. I have also used smooth rollers, with which it is essential to have the bearings work with perfect smoothness, but which, when they work perfectly, we would expect to empty the tissues more completely, and with less bruising. It may be questioned, however, if a certain amount of bruising, enough to secure the free outflow of serous exudate, practically washing out the trachomatous matter as the tissue is gone over the second or third time, is not an important part of the treatment. So that on the whole the corrugated rollers are probably the better.

In conclusion, let me urge all who may have to deal with trachoma in its marked and characteristic manifestations, to put the patient under ether and roll the lids freely, squeezing out all the exudate possible, and going over the whole conjunctiva two or three times.

Do not fear the apparent violence done the tissues. If the rolls turn properly, you cannot tear the conjunctiva; you will preserve all the epithelial surface there is to start with, and secure the minimum of cicatricial contraction. And in favorable cases, a cure is accomplished by a single operation, sometimes without after-treatment.

## Figures and Tables

**Figure f1:**